# GPR55 deletion increases anxiety- and depression-like behaviors and modifies amygdalar GABA_A_α2 expression

**DOI:** 10.3389/fphar.2026.1766492

**Published:** 2026-09-03

**Authors:** María S. García-Gutiérrez, Lucía Illescas, Ani Gasparyan, Jorge Manzanares

**Affiliations:** 1 Instituto de Neurociencias, Universidad Miguel Hernández-CSIC, Alicante, Spain; 2 Red de Investigación en Atención Primaria de Adicciones, Instituto de Salud Carlos III, MICINN and FEDER, Madrid, Spain; 3 Instituto de Investigación Sanitaria y Biomédica de Alicante (ISABIAL), Alicante, Spain; 4 Hepatic and Intestinal Immunobiology Group, Dpto. Medicina Clínica and Instituto IDIBE, Universidad Miguel Hernández, Alicante, Spain

**Keywords:** anxiety, depression, GABA_A_, GPR55, knockout mice, stress

## Abstract

**Introduction:**

GPR55 has attracted attention for its anti-inflammatory, neuroprotective, and neurotransmitter-modulating properties, suggesting a role in mood regulation. Here, we aimed to clarify the contribution of GPR55 to emotional responses.

**Methods:**

Male and female GPR55 knockout (GPR55KO) and wild-type (WT) C57BL/6J mice were evaluated in the light-dark box, elevated plus maze, social interaction and tail suspension tests. Gene expression of the GABA_A_ receptor α2 and γ2 subunits was measured in the amygdala (AMY). A separate cohort underwent 30 min of restraint stress to assess hypothalamic-pituitary-adrenal (HPA) axis markers, including the expression of the Crf and Nrc3c1 genes in the paraventricular nucleus (PVN) and in the hippocampus.

**Results:**

GPR55 deletion increased anxiety- and depressive-like behaviors across all the behavioral paradigms evaluated. GABA_A_α_2_ gene expression in the AMY was elevated in GPR55KO mice of both sexes, while GABA_A_γ_2_ expression was not influenced by genotype. Restraint stress increased *Crf* and reduced *Nr3c1* similarly across genotypes. Notably, male GPR55KO mice displayed lower basal *Crf* levels than male controls.

**Discussion:**

Together, these findings indicate that loss of GPR55 heightens emotional vulnerability and alters specific GABAergic markers in the AMY while preserving HPA axis-related transcriptional response to acute stress. These results support GPR55 as a potential target for the modulation of anxiety- and depression-related states.

## 1 Introduction

G protein–coupled receptors (GPCRs) comprise a significant class of pharmacological targets that regulate multiple physiological and neuropsychiatric processes. Among them, GPR55 has emerged as a receptor of interest due to its broad distribution in the central nervous system (CNS), including limbic and cortical regions (Pertwee, 2007; Sylantyev et al., 2013). Although initially considered an atypical cannabinoid receptor, GPR55 exhibits a distinct pharmacological profile and engages signaling mechanisms different from those of classical cannabinoid receptors, supporting a unique role in the CNS.

GPR55 has been proposed to play key roles in neuroprotection, neurotransmission, and cognitive processes such as learning and memory (Marichal-Cancino et al., 2018; Wang et al., 2022; Manduca et al., 2024; Sun T. et al., 2024). Growing evidence also implicates this receptor in behavioral and molecular processes linked to mood regulation. For example, altered GPR55 expression has been reported in the dorsolateral prefrontal cortex of patients who died by suicide (Garcia-Gutierrez et al., 2018). Similarly, animal models have shown that exposure to chronic stress paradigms affects brain GPR55 expression (Marco et al., 2014; Shi et al., 2017; Huang et al., 2021). Pharmacological modulation of GPR55 by selective agonists has been associated with anti-inflammatory (Saliba et al., 2018; Apweiler et al., 2024; Sun L. et al., 2024) and neuroprotective actions (Kallendrusch et al., 2013; Hill et al., 2019; Shen et al., 2022), as well as antidepressant- and anxiolytic-like effects in rodent models (Rahimi et al., 2015; Shi et al., 2017; Wrobel et al., 2020; Zhang et al., 2024). Conversely, inhibition of GPR55, through antagonists or gene silencing, has been linked to increased anxiety-like behaviors (Rahimi et al., 2015; Shi et al., 2017; Vazquez-Leon et al., 2021; Sanchez-Zavaleta et al., 2025). In parallel, GPR55 signaling has been implicated in the modulation of monoaminergic and glutamatergic transmission, and in stress-related plasticity, mechanisms that are central to the pathophysiology of anxiety and depressive disorders (Marichal-Cancino et al., 2017; He et al., 2024).

Despite this growing interest, the functional role of GPR55 in emotional regulation remains insufficiently defined, and available findings show variability across experimental models and brain regions. A key limitation is the scarcity of studies examining the behavioral consequences of GPR55 loss-of-function together with downstream molecular adaptations. It remains unclear whether the absence of GPR55 affects the limbic GABAergic system, particularly within the amygdala (AMY), a central region for emotional processing. Moreover, the potential influence of sex on behavioral or molecular alterations associated with GPR55 signaling has received limited attention, despite its recognized relevance in affective disorders.

To address these gaps, we evaluated anxiety- and depression-like behaviors in male and female GPR55 knockout (GPR55KO) mice and quantified GABAergic markers in the AMY. Additionally, we examined the expression of the hypothalamic–pituitary–adrenal (HPA) axis–related genes following acute restraint stress. This combined behavioral and molecular approach aimed to clarify GPR55’s contribution to emotional regulation and identify potential sex-related differences relevant to its neuropharmacological profile.

## 2 Materials and methods

### 2.1 Mice

GPR55KO mice were kindly provided by Shanta Persaud (King’s College London, United Kingdom) and generated as previously described (Liu et al., 2016; Ruz-Maldonado et al., 2020). Briefly, a targeted mutation was introduced in 129SvEvBrd-derived embryonic stem cells by inserting a cassette that replaced a portion of exon 2 of the *Gpr55* gene, thereby disrupting the entire GPR55 coding sequence. Homologous recombination was confirmed by Southern blot analysis using probes internal to the targeting vector on the 5′ side and external to the vector on the 3′ side. Following blastocyst injection, chimeric mice were bred to C57BL/6J mice to generate F1 heterozygous animals.

Genotypes were confirmed by polymerase chain reaction (PCR) using the following primers: GPR55KO F1: 5′ TCT GGA TTC ATC GAC T 3′; GPR55KO R1: 5′ TCC ACA ATC AAG CTG 3′; GPR55KO R2: 5′ GTC ACC CAT CCA GGT GAT 3′. PCR conditions consisted of an initial denaturation at 94 °C for 3 min, followed by 35 cycles at 94 °C for 1 min, 55 °C for 1 min, and 72 °C for 1 min, and a final extension at 72 °C for 10 min. Amplicons of 207 bp (F1+R1) and 299 bp (F1+R2) correspond to the wild-type and mutated alleles, respectively.

The colony was maintained by periodic outcrossing to C57BL6/J mice (Charles River, Spain) every 10 generations to preserve the genetic background. Heterozygous breeders were intercrossed to obtain wild-type (WT) and homozygous GPR55KO littermates. WT and GPR55KO male and female mice (6 weeks old, 20–25 g) were used for experiments. Mice were housed in groups of five per cage (40 cm × 25 cm × 22 cm) under controlled temperature (21 °C ± 2 °C), humidity (60% ± 10%), and a 12-h light/dark cycle (lights on from 8:00 a.m. to 8:00 p.m.).

All procedures complied with Spanish regulations (RD 1201/2005, Law 32/2007) and the EU Directive 2010/63/EU.

The sample sizes (n) for each experimental group included in the study are summarized in Tables 1 and 2.

**TABLE 1:** Sample sizes (n) for the behavioral tests and GABA-related qPCR studies.

Experiment	♂WT	♂GPR55KO	♀WT	♀GPR55KO	Total
OF	12	12	12	12	48
LDB	10	12	10	10	42
EPM	10	12	10	10	42
SI*	5	5	5	5	20
TST	9	10	9	9	37
qPCR GABA_A_α_2_	10	12	9	10	41
qPCR GABA_A_γ_2_	10	11	9	10	40

* For SI, n refers to the number of interacting pairs.

OF, open field test; LDB, light-dark box test; EPM, elevated plus maze test; SI, social interaction test; TST, tail suspension test; qPCR, real time PCR.

**TABLE 2:** Sample sizes (n) for the restraint stress cohort used for HPA axis gene expression analyses.

Group	qPCR *Crf*	qPCR *Nr3c1*
♂WT Non-stress	11	11
♂WT Stress	11	9
♂GPR55KO Non-stress	8	10
♂GPR55KO Stress	11	12
♀WT Non-stress	10	10
♀WT Stress	10	9
♀GPR55KO Non-stress	9	9
♀GPR55KO Stress	9	9

*Crf*, gene encoding corticotropin-releasing factor; *Nr3c1*, gene encoding the glucocorticoid receptor.

### 2.2 Open field test

The open field (OF) consisted of a transparent acrylic arena (25 cm × 25 cm × 25 cm) with a white floor (Garcia-Gutierrez and Manzanares, 2011; Torregrosa et al., 2025). Each mouse was placed gently in the center of the apparatus, and its spontaneous locomotor activity was recorded for 20 min using a video camera. Behavioral tracking was performed with the SMART software system (version 2.5.3, Panlab, Barcelona, Spain).

### 2.3 Behavioral procedures for emotional reactivity

#### 2.3.1 Light-dark box test

The light-dark box (LDB) test was used to assess anxiety-like behavior based on rodents’ natural aversion to brightly illuminated areas (Crawley and Goodwin, 1980; Garcia-Gutierrez and Manzanares, 2011). The apparatus consisted of two plastic chambers (20 cm × 20 cm × 15 cm): one transparent and open (light compartment) and one opaque and covered (dark compartment), connected by a dark plastic tunnel (4 cm). The light compartment was illuminated with a 60 W lamp positioned 25 cm above it. Each mouse was placed in the light compartment facing the tunnel, and behavior was recorded for 5 min. The time spent in the light compartment and the number of transitions between compartments were measured. A transition was defined as entry with both forepaws and at least one hind paw.

#### 2.3.2 Elevated plus maze test

The elevated plus maze (EPM) test assesses anxiety-like behavior by measuring avoidance of open and high areas (Lister, 1987; Garcia-Gutierrez and Manzanares, 2011). The apparatus consisted of two open and two perpendicular closed arms, elevated 50 cm from the floor. The union of the four arms formed a central square platform (5 cm × 5 cm). Under standard lighting conditions (100 lux), each mouse was placed in the center of the apparatus, facing a closed arm. The total time spent in open arms and the number of transitions between arms were recorded during a 5-min session. Transitions were counted when both forepaws and at least one hind paw entered a different arm.

#### 2.3.3 Social interaction test

The social interaction (SI) test assesses anxiety-like behavior by exposing mice to two anxiogenic stimuli: a novel environment and an unfamiliar conspecific (Uriguen et al., 2004). Pairs of mice, matched by genotype and sex, from different home cages were placed together on a small plastic cage (20 cm × 40 cm × 10 cm) with a cardboard lid and fresh wood litter on the floor. The time spent in social interaction and the number of interactions (sniffing, following, grooming, kicking, crawling under or over the partner, and touching or nearly touching the partner’s face) were measured over 5 min.

#### 2.3.4 Tail suspension test

In the tail suspension test (TST), mice were individually suspended by their tails at the edge of a lever above the tabletop, positioned 35 cm from the surface, and fixed with adhesive tape approximately 1–2 cm from the tail tip (Vaugeois et al., 1997; Garcia-Gutierrez et al., 2010). The immobility time was measured for 6 min. In this paradigm, mice exhibit escape-oriented behaviors interspersed with temporally increasing episodes of immobility.

### 2.4 Acute restraint stress

GPR55KO and WT mice were randomly assigned to a non-stress or acute restraint stress (ARS) group (Garcia-Gutierrez and Manzanares, 2011). The control group (non-stress) was left undisturbed in their home cages throughout the assay and was handled only for routine housekeeping tasks. For ARS, mice were subjected to 30 min of restraint stress by placing them in cylindrical acrylic tubes (10 cm × 3 cm × 3 cm). After restraint, mice were released and returned to their home cages.

### 2.5 Gene expression analysis by qPCR

qPCR was performed to assess whether deletion of GPR55 modifies basal relative gene expression of GABA_A_α_2_ and GABA_A_γ_2_ in the AMY of mice from the behavioral cohort. In the cohort exposed to ARS, the potential impact of GPR55 deletion on stress responsiveness was evaluated by measuring the HPA axis-related markers. Specifically, expression levels of the *Crf* gene (encoding corticotropin-releasing factor) in the paraventricular nucleus (PVN) and of the *Nr3c1* gene (encoding the glucocorticoid receptor) in the hippocampus (HIP) were analyzed.

Brains from the ARS were collected 2.5 h after the restraint procedure ended to capture stress-induced changes in gene expression. In contrast, brains from the behavioral cohort were collected the day following completion of the behavioral battery, allowing gene expression to reflect basal conditions rather than acute transcriptional responses to behavioral testing.

The brains were removed from the skull, frozen on dry ice, and stored at −80 °C. Brain sections measuring 500 μm were cut at various levels, including the regions of interest (AMY, HIP and PVN), using a cryostat, following the Paxinos and Franklin Mouse Brain Atlas (Franklin, 2001). The sections were then mounted on slides and stored at −80 °C. A section from each level was microdissected using the method of Palkovits (Palkovits, 1983).

Total RNA from brain punctures was isolated using TRI Reagent (ThermoFisher Scientific, Madrid, Spain). cDNA was generated using a High-Capacity cDNA Reverse Transcription Kit with RNase inhibitor (ThermoFisher Scientific, Madrid, Spain).

Gene expression was quantified using TaqMan probes for *Crf* (Mm01293920_s1), *Gabra2* (Mm00433435_m1), *Gabrg2* (Mm00433489_m1), and *Nr3c1* (Mm00435874_m1), which use a specific fluorescent dye for double-stranded DNA. All reactions were run in duplicate on a StepOne Plus Real-Time PCR System (ThermoFisher Scientific, Madrid, Spain). 18S rRNA (Mm03928990_g1) was used as the reference gene. Briefly, data for each target gene were normalized to the endogenous reference gene, and the changes in target gene abundance were determined using the 2^−^ΔΔCt method (Livak and Schmittgen, 2001).

### 2.6 Statistical analysis

Statistical analyses were performed using SigmaPlot 11.0 (Systat Software Inc., Chicago, IL, United States of America). Data are presented as mean ± SEM, and statistical significance was set at p < 0.05. Normality (Shapiro–Wilk) and homogeneity of variance (Levene’s test) were assessed before analysis. When assumptions were reasonably met, parametric analyses were conducted using two-way ANOVA (genotype × sex), followed by Tukey’s *post hoc* test when appropriate.

Datasets that did not satisfy parametric assumptions and were not improved by transformation were analyzed using non-parametric procedures, specifically Kruskal–Wallis one-way ANOVA on ranks followed by Dunn’s *post hoc* test when required. Statistical procedures were selected according to data distribution characteristics and the suitability of each method for the corresponding dataset.

## 3 Results

### 3.1 Open field test

**FIGURE 1:**
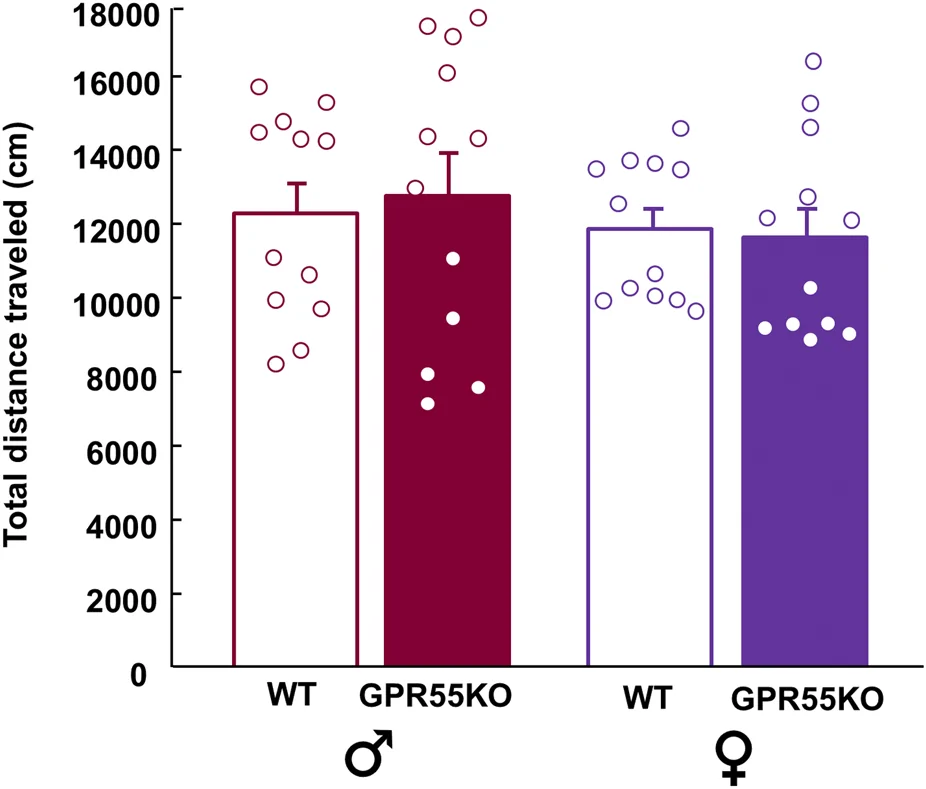
Open field performance in WT and GPR55KO mice. Total distance traveled during the open field test. Data are expressed as mean ± SEM (n = 12 per group). Statistical analysis was performed using Kruskal–Wallis one-way ANOVA on ranks.

No significant group differences were detected in total distance traveled (Figure 1; Kruskal–Wallis one-way ANOVA on ranks test, H = 0.99, df = 3, p = 0.804) (n = 12/group), indicating that locomotor activity was similar across genotypes and sexes.

### 3.2 Light dark box test

**FIGURE 2:**
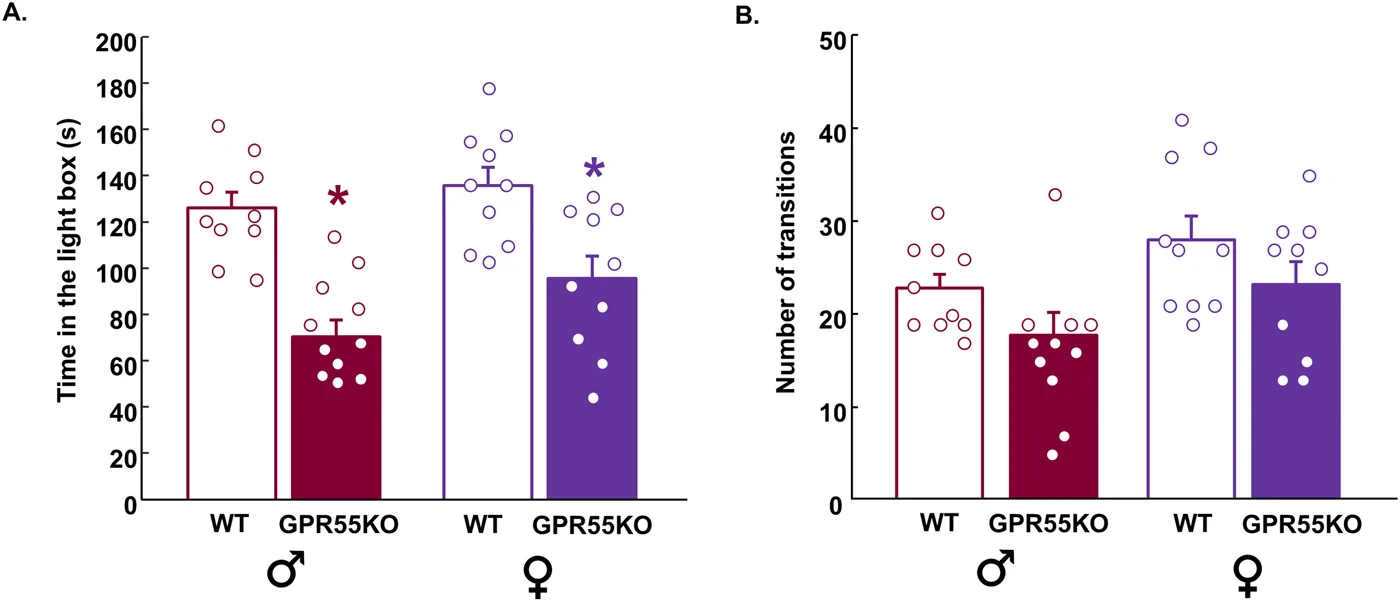
Light–dark box performance in WT and GPR55KO mice. **(A)** Time spent in the light compartment and **(B)** number of transitions between the light and dark compartments. Data are expressed as mean ± SEM (n = 9–12 per group). Statistical analysis was performed using two-way ANOVA followed by Tukey’s *post hoc* test. *p < 0.05 vs. WT of the same sex.

GPR55KO mice spent less time in the light compartment compared with WT mice, an effect observed in both sexes (Figure 2A; Two-Way ANOVA followed by Tukey’s *post hoc* test, genotype F_(1,38)_ = 36.231, p < 0.001; sex F_(1,38)_ = 4.804, p = 0.035; genotype x sex F_(1,38)_ = 0.961, p = 0.333) (n = 9–12/group).

In contrast, the number of transitions between compartments showed significant main effects of genotype and sex; however, *post hoc* comparisons did not reveal significant pairwise differences among groups (Figure 2B; Two-Way ANOVA followed by Tukey’s *post hoc* test, genotype F_(1,38)_ = 4.599, p = 0.038; sex F_(1,38)_ = 5.376, p = 0.026; genotype x sex F_(1,38)_ = 0.003, p = 0.957) (n = 9–12/group). Overall, transition frequency remained comparable across groups.

### 3.3 Elevated plus maze test

**FIGURE 3:**
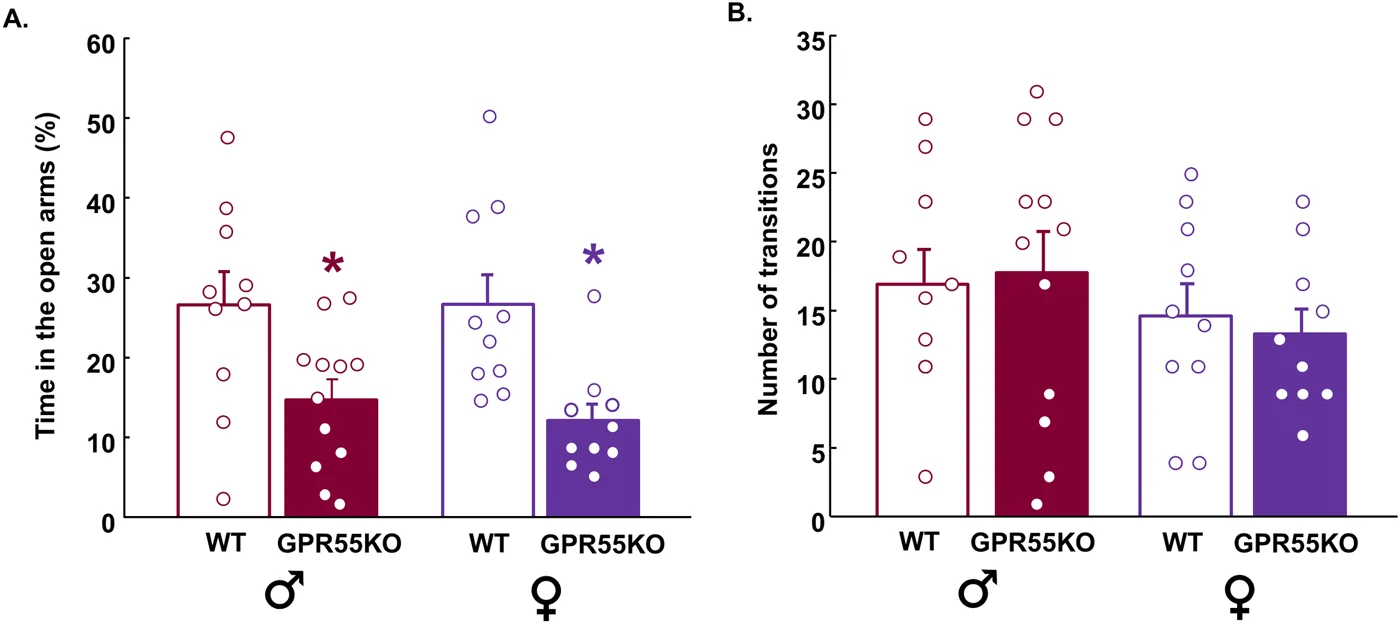
Elevated plus maze performance in WT and GPR55KO mice. **(A)** Time spent in the open arms and **(B)** number of transitions between arms. Data are expressed as mean ± SEM (n = 10–12 per group). Statistical analysis was performed using two-way ANOVA followed by Tukey’s *post hoc* test. *p < 0.05 vs. WT of the same sex.

In the EPM, GPR55KO exhibited a marked reduction in the time spent in the open arms compared relative to their WT counterparts, with no influence of sex on this effect (Figure 3A; Two-Way ANOVA followed by Tukey’s *post hoc* test, genotype F_(1,38)_ = 16.931, p < 0.001; sex F_(1,38)_ = 0.159, p = 0.692; genotype x sex F_(1,38)_ = 0.167, p = 0.685) (n = 10–12/group).

Arms transitions, however, were comparable across groups, as neither genotype nor sex affected the number of transitions (Figure 3B; Two-Way ANOVA followed by Tukey’s *post hoc* test, genotype F_(1,38)_ = 0.00795, p = 0.929; sex F_(1,38)_ = 1.788, p = 0.189; genotype x sex F_(1,38)_ = 0.181, p = 0.673) (n = 10–12/group), indicating preserved exploratory activity during the test.

### 3.4 Social interaction test

**FIGURE 4:**
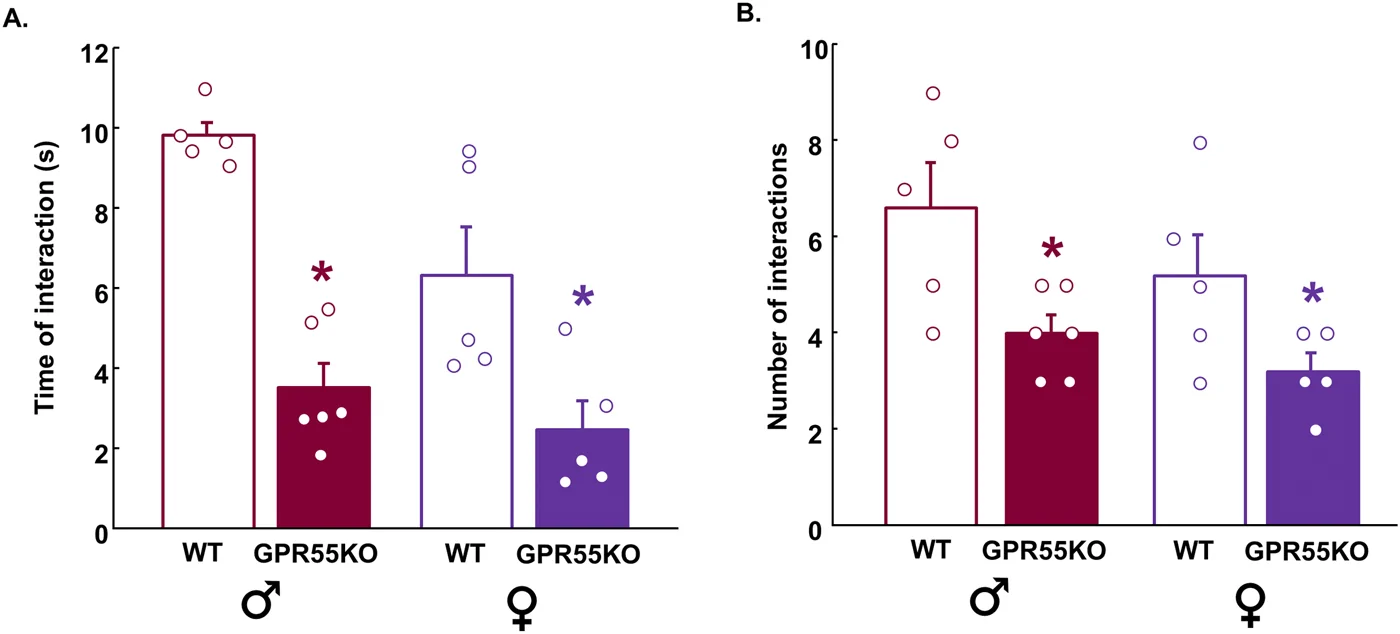
Social interaction test performance in WT and GPR55KO mice. **(A)** Time of interaction and **(B)** number of interactions. Data are expressed as mean ± SEM (n = 5 interacting pairs per group). Statistical analysis was performed using two-way ANOVA followed by Tukey’s *post hoc* test. *p < 0.05 vs. WT of the same sex.

GPR55KO mice showed a significant reduction in both the time spent interacting (Figure 4A; Two-way ANOVA followed by Tukey’s *post hoc* test, genotype F_(1,17)_ = 43.629, p < 0.001; sex F_(1,17)_ = 8.695, p = 0.009; genotype x sex F_(1,17)_ = 2.537, p = 0.130) (n = 5/group) and number of interactions compared to WT mice (Figure 4B; Two-way ANOVA followed by Tukey’s *post hoc* test, genotype F_(1,17)_ = 12.093, p = 0.003; sex F_(1,17)_ = 2.766, p = 0.115; genotype x sex F_(1,17)_ = 0.206, p = 0.656) (n = 5/group). Overall, GPR55KO mice displayed reduced social interaction compared to WT controls.

### 3.5 Tail suspension test

**FIGURE 5:**
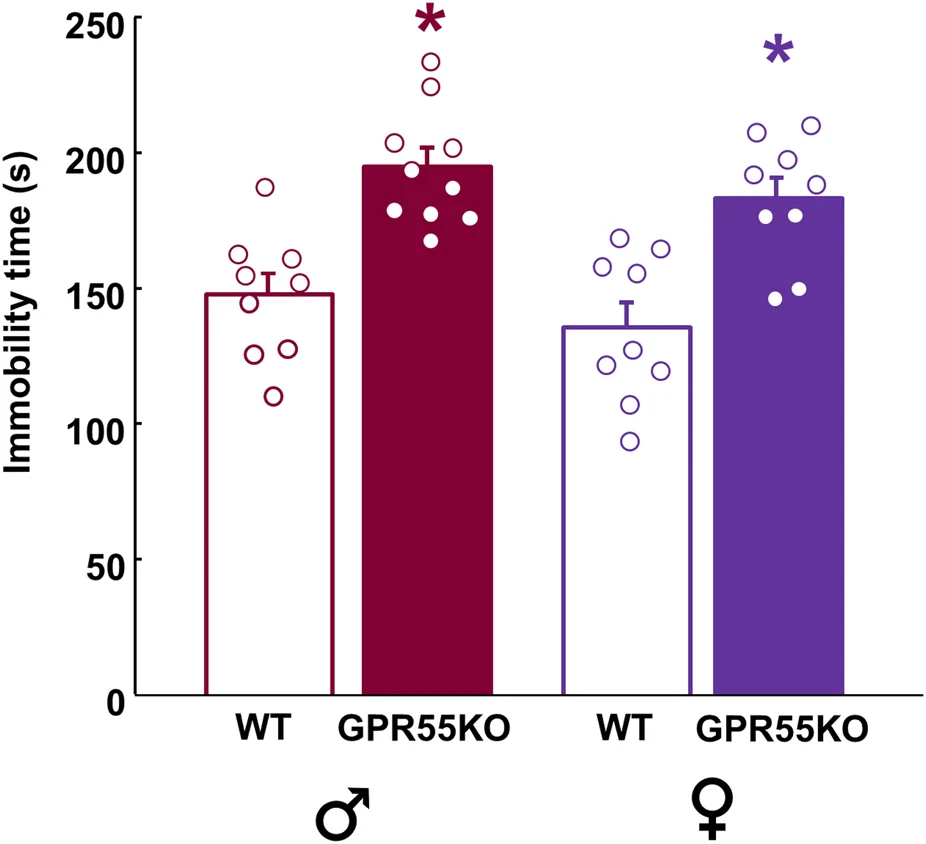
Tail suspension test performance in WT and GPR55KO mice. Immobility time during the tail suspension test. Data are expressed as mean ± SEM (n = 9–10 per group). Statistical analysis was performed using two-way ANOVA followed by Tukey’s *post hoc* test. *p < 0.05 vs. WT of the same sex.

In the TST, GPR55KO mice displayed a pronounced increase in immobility time compared with WT mice, indicating an enhanced depressive-like phenotype (Figure 5; Two-way ANOVA followed by Tukey’s *post hoc* test, genotype F_(1,33)_ = 36.584, p < 0.001; sex F_(1,33)_ = 2.325, p = 0.137; genotype x sex F_(1,33)_ = 0.000731, p = 0.979) (n = 9–10/group). Thus, the effect of GPR55 deletion on immobility was consistent across sexes.

### 3.6 qPCR analysis of GABAergic gene expression

**FIGURE 6:**
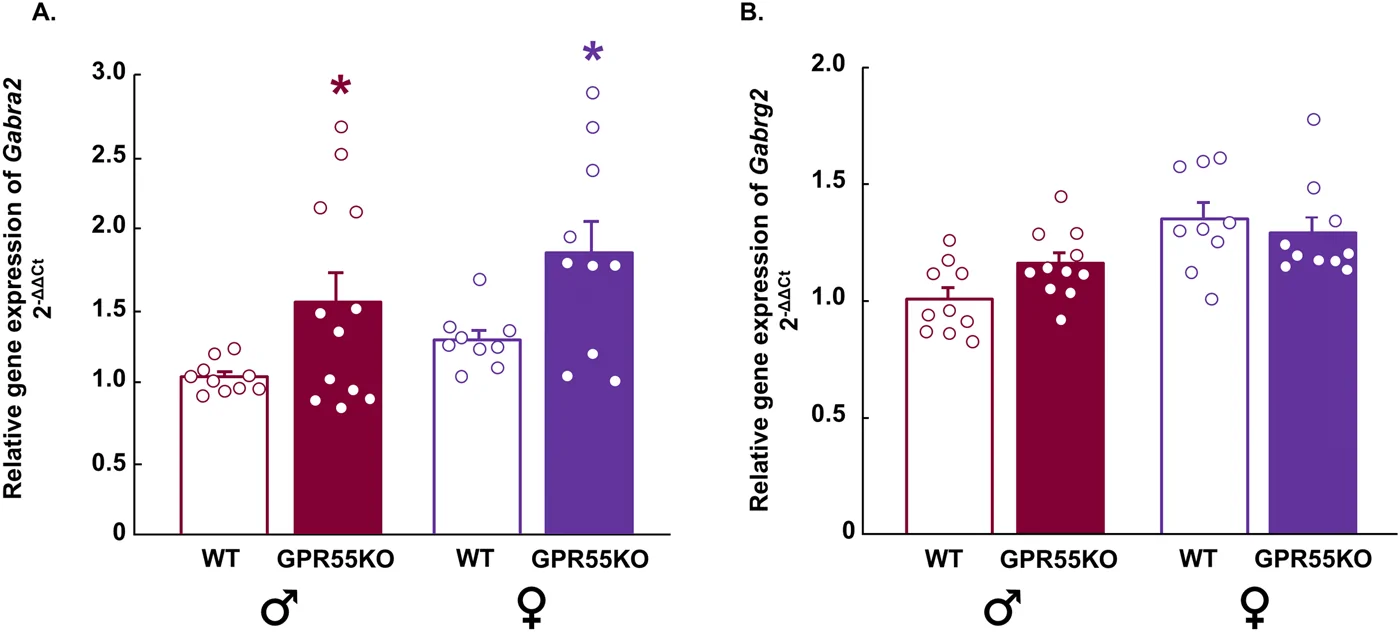
GABAergic gene expression in the amygdala of WT and GPR55KO mice. **(A)** Relative gene expression of the GABA_A_ receptor α2 subunit. **(B)** Relative gene expression of the GABA_A_ receptor γ2 subunit. Data are expressed as mean ± SEM (n = 10–12 per group). Statistical analysis was performed using two-way ANOVA followed by Tukey’s *post hoc* test. *p < 0.05 vs. WT of the same sex.

Analysis of GABA_A_α_2_ in the AMY revealed that GPR55KO mice exhibited significantly higher expression levels compared to their WT counterparts, independently of sex (Figure 6A; Two-way ANOVA followed by Tukey’s *post hoc* test, genotype F_(1,37)_ = 11.386, p = 0.002; sex F_(1,37)_ = 3.259, p = 0.079; genotype x sex F_(1,37)_ = 0.0679, p = 0.796) (n = 10–12/group).

In contrast, GABA_A_γ_2_ expression was not affected by GPR55 deletion, as no significant genotype-dependent differences were detected. Interestingly, a robust main effect of sex was observed, indicating overall differences in γ2 expression between males and females (Figure 6B; Two-way ANOVA followed by Tukey’s *post hoc* test, genotype F_(1,36)_ = 0.696, p = 0.410; sex F_(1,36)_ = 17.424, p < 0.001; genotype x sex F_(1,36)_ = 3.517, p = 0.069) (n = 10–11/group).

### 3.7 qPCR analysis of HPA gene expression

**FIGURE 7:**
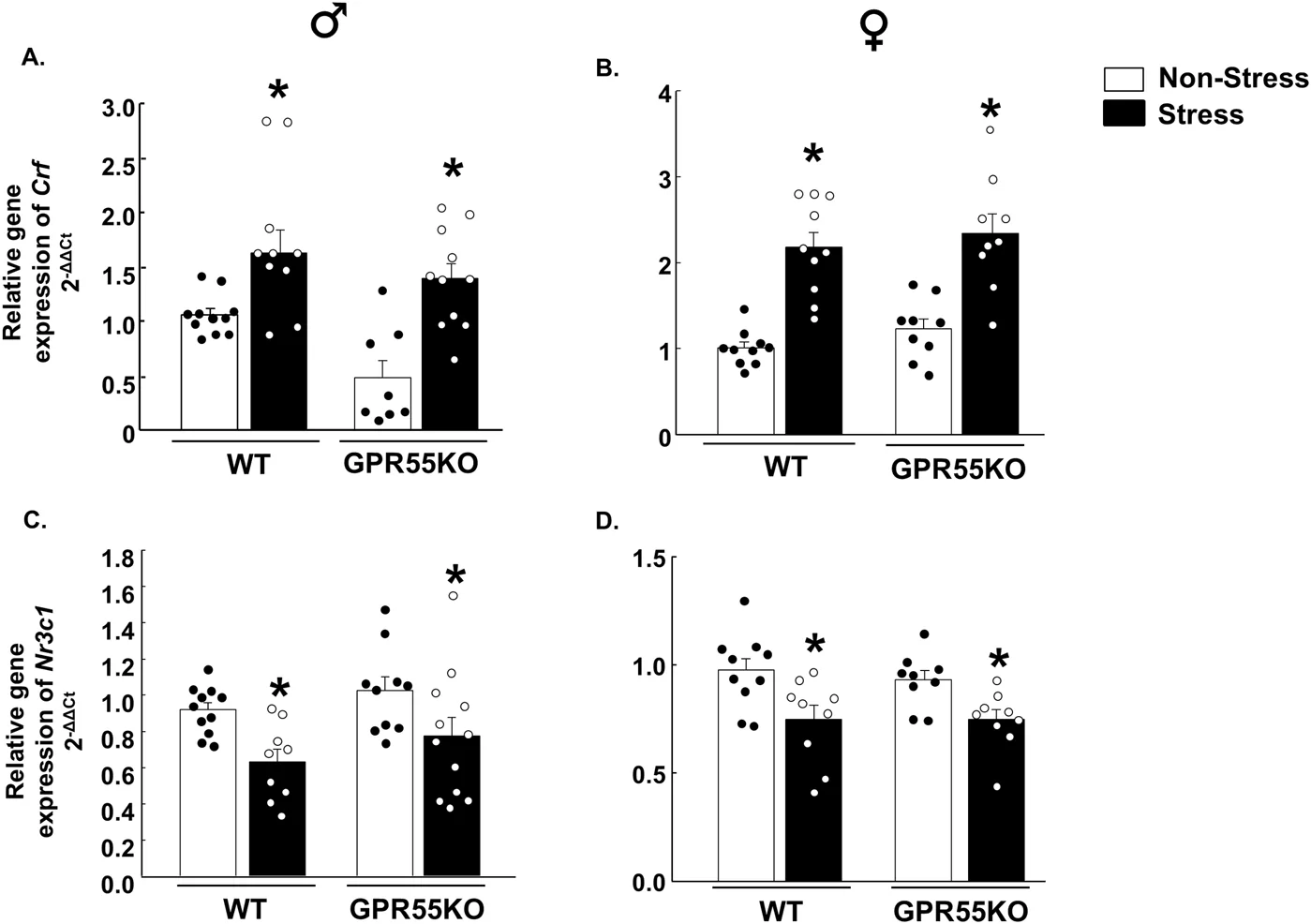
HPA axis–related gene expression in response to acute restraint stress. **(A,B)** Relative *Crf* gene expression in the paraventricular nucleus (PVN) of males **(A)** and females **(B) (C,D)** Relative *Nr3c1* gene expression in the hippocampus of males **(C)** and females **(D)** Data are expressed as mean ± SEM (n = 8–12 per group). Statistical analysis was performed using two-way ANOVA followed by Tukey’s *post hoc* test. *p < 0.05 vs. non-stressed animals of the same genotype and sex.

In males, ARS significantly increased *Crf* expression in the PVN, independently of genotype. Basal *Crf* levels were lower in GPR55KO mice, whereas the magnitude of the stress-induced response was comparable between genotypes (Figure 7A; Two-way ANOVA followed by Tukey’s *post hoc* test, genotype F_(1,37)_ = 7.121, p = 0.011; stress F_(1,37)_ = 23.366, p < 0.001; genotype x stress F_(1,37)_ = 1.253, p = 0.270) (n = 8–11/group).

In females, restraint stress markedly increased *Crf* expression independently of genotype, with no evidence of genotype-dependent effects (Figure 7B; Two-way ANOVA followed by Tukey’s *post hoc* test, genotype F_(1,34)_ = 1.572, p = 0.219; stress F_(1,34)_ = 54.999, p < 0.001; genotype x stress F_(1,34)_ = 0.0323, p = 0.858) (n = 9–10/group).

Additional three-way ANOVA analysis of *Crf* expression revealed a significant sex × genotype interaction (F_1_,_71_ = 7.625, p = 0.007), whereas the sex × genotype × stress interaction was not significant (F_1_,_71_ = 0.830, p = 0.365), supporting the interpretation that acute stress response were preserved across phenotype while basal *Crf* expression differed according to sex and genotype.

In males, stress significantly reduced *Nr3c1* expression in both genotypes (Figure 7C; Two-way ANOVA followed by Tukey’s *post hoc* test, genotype F_(1,38)_ = 2.537, p = 0.119; stress F_(1,38)_ = 11.852, p = 0.001; genotype x stress F_(1,38)_ = 0.0541, p = 0.817) (n = 11–12/group).

A similar pattern was observed in females, where restraint stress also produced a significant decrease in *Nr3c1* expression independently of genotype (Figure 7D; Two-way ANOVA followed by Tukey’s *post hoc* test, genotype F_(1,33)_ = 0.201, p = 0.657; stress F_(1,33)_ = 14.788, p < 0.001; genotype x stress F_(1,33)_ = 0.170, p = 0.683) (n = 9–10/group).

## 4 Discussion

This study provides evidence supporting a role for GPR55 in modulating emotional behavior. Genetic deletion of GPR55 increased anxiety- and depressive-like phenotypes across multiple behavioral paradigms. In addition, loss of GPR55 was associated with alterations in the expression of the GABA_A_ receptor α2 subunit, closely related to anxiety regulation. In contrast, GPR55 deletion did not alter the acute stress-induced expression of HPA axis-related markers. Nevertheless, under basal conditions, GPR55 deletion reduced *Crf* expression in the PVN in males, highlighting a specific sex-related difference that warrants further investigation.

GPR55 is widely expressed throughout the CNS, including corticolimbic regions critically involved in emotional regulation and the pathophysiology of psychiatric disorders (Pertwee, 2007; Sylantyev et al., 2013). Although previous studies have examined the functional role of GPR55, further investigation is needed to clarify its contribution to emotional behavior, particularly regarding potential sex-related differences (Hill et al., 2019; Wang et al., 2022; Manduca et al., 2024; Sun L. et al., 2024; Sun T. et al., 2024). In this study, the use of a comprehensive battery of behavioral paradigms revealed increases in anxiety-like responses in the LDB, EPM and SI tests in GPR55KO mice. Moreover, genetic deletion of the GPR55 receptor enhanced behavioral despair, as shown by increased immobility time in the TST. Importantly, the absence of GPR55 did not affect the basal locomotor activity, as demonstrated by the OF, where no differences in total distance traveled were recorded. Total distance in the OF is a widely used parameter to assess general locomotor activity in rodents (Seibenhener and Wooten, 2015), supporting the conclusion that the behavioral alterations detected across the different parameters are not attributable to changes in motor function.

These results are consistent with previous pharmacological studies showing anxiolytic and antidepressant-like effects following GPR55 activation (Rahimi et al., 2015; Shi et al., 2017; Wrobel et al., 2020; Zhang et al., 2024), whereas GPR55 antagonism or inhibition produces the opposite behavioral outcomes (Rahimi et al., 2015; Shi et al., 2017; Vazquez-Leon et al., 2021; Sanchez-Zavaleta et al., 2025). Collectively, the present findings support a critical role for GPR55 in modulating emotional responses and reinforce its potential as a pharmacological target for affective disorders.

The GABAergic system plays a fundamental role in the regulation of emotional behavior, and disturbances in inhibitory neurotransmission are closely associated with anxiety and mood disorders (Luscher et al., 2011; Nuss, 2015). In particular, the α2 and γ2 subunits of the GABA_A_ receptor are critically involved in anxiolytic responses and represent key molecular targets of benzodiazepines (Elgarf et al., 2018). In this study, loss of GPR55 was associated with significant upregulation of GABA_A_α_2_ subunit gene expression in the AMY, independently of sex.

Although direct molecular interactions between GPR55 and GABA_A_ receptors have not been extensively characterized, previous evidence suggests that GPR55 signaling modulates neuronal excitability and synaptic plasticity, processes that critically rely on inhibitory GABAergic circuits (Musella et al., 2017; Sanchez-Zavaleta et al., 2022). In this context, the increased expression of the GABA_A_α_2_ subunit observed in the AMY of GPR55KO mice suggests that GPR55 deletion is associated with alterations in GABAergic markers within limbic circuits involved in emotional processing. However, further studies will be required to determine the functional significance of these transcriptional changes.

Finally, the HPA axis represents a core neuroendocrine system mediating the physiological and behavioral responses to stress, and its dysregulation has been consistently implicated in anxiety and depressive disorders (Holsboer, 1989; de Kloet et al., 2005; Menke, 2024). In the present study, genetic deletion of GPR55 did not modify the acute stress-induced transcriptional response of HPA axis-related markers, since WT and GPR55KO mice displayed a comparable increase in *Crf* expression in the PVN, accompanied by a reduction in *Nr3c1* expression in the HIP following ARS. Notably, these molecular changes are consistent with well-established transcriptional signatures of ARS, indicating that the expected stress-induced changes in the HPA axis-related gene expression occurred in both genotypes (Noguchi et al., 2010; Garcia-Gutierrez and Manzanares, 2011).

However, under basal conditions, male GPR55KO mice showed reduced *Crf* expression in the PVN compared with WT controls. Although the biological significance of this finding remains unclear, this alteration suggests that GPR55 deletion may influence the basal *Crf* regulation in a sex-related manner. Such changes in basal *Crf* expression could reflect an altered stress set point that contributes to increased vulnerability to anxiety- and depression-like phenotypes, rather than reflecting alterations in the acute stress-induced gene expression profile. Further studies incorporating endocrine measurements will be necessary to better understand the role of GPR55 in HPA axis regulation.

In summary, this study identifies GPR55 as a potential modulator of emotional behavior, whose genetic deletion leads to increased anxiety- and depression-like phenotypes accompanied by alterations in amygdalar GABA_A_α2 expression while preserving the appropriate transcriptional response of the HPA axis to acute stress. These findings suggest that GPR55 contributes to the fine-tuning of inhibitory and stress-related circuits that regulate emotional reactivity.

Nevertheless, several limitations should be acknowledged. The use of a global knockout model limits conclusions about region- or cell-type–specific mechanisms, and future studies incorporating rescue approaches or circuit-specific manipulations will be necessary to establish causal relationships. Moreover, molecular analyses were restricted to gene expression levels, and the functional relevance of these changes remains to be established. Additional brain regions and molecular pathways beyond those examined in the present study may also contribute to the emotional phenotype associated with GPR55 deletion. Furthermore, the relatively small sample size used in the social interaction test may have reduced the sensitivity to detect subtle interaction effects between sex and genotype. In addition, stress-related experiments focused on an acute paradigm, leaving the role of GPR55 under chronic stress conditions unresolved. Despite these limitations, the convergence of behavioral and molecular findings supports the notion that GPR55 may represents a pharmacologically relevant target in anxiety- and mood-related disorders, deserving further investigation using complementary genetic and pharmacological approaches.

## Data Availability

The raw data supporting the conclusions of this article will be made available by the authors, without undue reservation.

## In memoriam

In memory of author Jorge Manzanares.
